# Smith-Lemli-Opitz syndrome: A pathophysiological manifestation of the Bloch hypothesis

**DOI:** 10.3389/fmolb.2023.1120373

**Published:** 2023-01-12

**Authors:** Amitabha Chattopadhyay, Ashwani Sharma

**Affiliations:** ^1^ CSIR-Centre for Cellular and Molecular Biology, Hyderabad, India; ^2^ Academy of Scientific and Innovative Research, Ghaziabad, India

**Keywords:** SLOS, Bloch hypothesis, cholesterol biosynthesis, 7-DHC, serotonin_1A_ receptor, cellular trafficking

## Abstract

The biosynthesis of cholesterol, an essential component of higher eukaryotic membranes, was worked out by Konrad Bloch (and Feodor Lynen) in the 1960s and they received the Nobel Prize around that time in recognition of their pioneering contributions. An elegant consequence of this was a hypothesis proposed by Konrad Bloch (the Bloch hypothesis) which suggests that each subsequent intermediate in the cholesterol biosynthesis pathway is superior in supporting membrane function in higher eukaryotes relative to its precursor. In this review, we discuss an autosomal recessive metabolic disorder, known as Smith-Lemli-Opitz syndrome (SLOS), associated with a defect in the Kandutsch-Russell pathway of cholesterol biosynthesis that results in accumulation of the immediate precursor of cholesterol in its biosynthetic pathway (7-dehydrocholesterol) and an altered cholesterol to total sterol ratio. Patients suffering from SLOS have several developmental, behavioral and cognitive abnormalities for which no drug is available yet. We characterize SLOS as a manifestation of the Bloch hypothesis and review its molecular etiology and current treatment. We further discuss defective Hedgehog signaling in SLOS and focus on the role of the serotonin_1A_ receptor, a representative neurotransmitter receptor belonging to the GPCR family, in SLOS. Notably, ligand binding activity and cellular signaling of serotonin_1A_ receptors are impaired in SLOS-like condition. Importantly, cellular localization and intracellular trafficking of the serotonin_1A_ receptor (which constitute an important determinant of a GPCR cellular function) are compromised in SLOS. We highlight some of the recent developments and emerging concepts in SLOS pathobiology and suggest that novel therapies based on trafficking defects of target receptors could provide new insight into treatment of SLOS.

## Cholesterol: An essential and abundant lipid in higher eukaryotes

Cholesterol, an essential lipid in higher eukaryotic cellular membranes, has fascinated scientists since it was first isolated in the late 18th century (for a historical perspective of the discovery of cholesterol, see Kumar and Chattopadhyay, 2016). It is the single most abundant lipid in the higher eukaryotic plasma membrane (Maxfield and van Meer, 2010) accounting for ∼30–50 mol% of the total lipid content (Mouritsen and Bagatolli, 2016), whereas it is absent in prokaryotes. A hallmark of cholesterol is its role in the organization and dynamics of cellular membranes (Mouritsen and Zuckermann, 2004; Maxfield and van Meer, 2010). More importantly, cholesterol is known to regulate structure, function, dynamics, endocytosis and trafficking of important membrane proteins such as G protein-coupled receptors (GPCRs) (Pucadyil and Chattopadhyay, 2004; Pucadyil and Chattopadhyay, 2006; Pucadyil and Chattopadhyay, 2007a; Paila and Chattopadhyay, 2010; Shrivastava et al., 2010; Oates and Watts, 2011; Goddard and Watts, 2012; Jafurulla and Chattopadhyay, 2013; Chattopadhyay, 2014; Jafurulla et al., 2014; Jafurulla et al., 2019; Sengupta and Chattopadhyay, 2015; Gimpl, 2016; Sengupta et al., 2018; Kiriakidi et al., 2019; Jakubík and El-Fakahany, 2021; Kumar and Chattopadhyay, 2021a; Sarkar and Chattopadhyay, 2022).

An interesting feature of cholesterol organization in biological membranes is its non-random distribution in domains (or pools) in biological and model membranes (sometimes termed as “rafts”) (Schroeder et al., 1995; Brown and London, 1998; Wood et al., 1999; Xu and London, 2000; Mukherjee and Maxfield, 2004; Lingwood and Simons, 2010; Chaudhuri and Chattopadhyay, 2011). These domains are believed to be crucial since cellular processes such as membrane sorting/trafficking (Simons and van Meer, 1988), and signal transduction (Simons and Toomre, 2000) have been attributed to such domains. In addition, host membrane cholesterol has been shown to have a profound role in the entry of intracellular pathogens (Pucadyil et al., 2004; Pucadyil and Chattopadhyay, 2007b; Viswanathan et al., 2015; Kumar et al., 2016; Sanders et al., 2021).

## Cholesterol biosynthesis and the Bloch hypothesis

**FIGURE 1:**
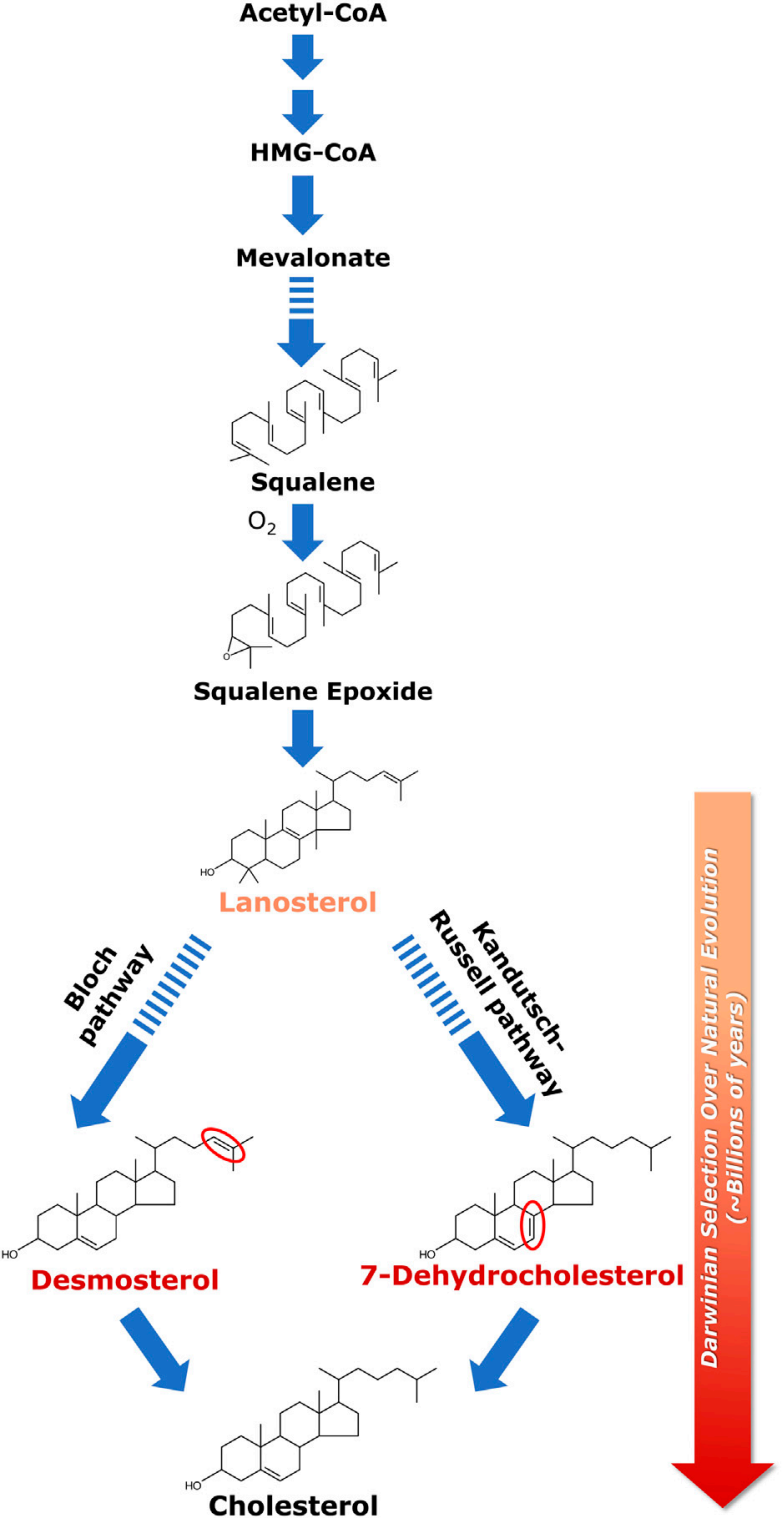
Biosynthesis of cholesterol and its evolutionary relevance. Cellular cholesterol biosynthesis commences with acetyl-CoA which gets converted to HMG-CoA in a two-step enzymatic reaction; HMG-CoA is then converted to lanosterol in a multi-step process. Squalene is the last linear molecule in the cholesterol biosynthesis pathway. Lanosterol (the first cyclic precursor in the cholesterol biosynthetic pathway) is the last common substrate for the two pathways of cholesterol biosynthesis, i.e., the Kandutsch-Russell pathway and the Bloch pathway. The difference between the two pathways lies in the iso-octyl chain in the intermediates. In the Kandutsch-Russell pathway, the intermediates have a saturated iso-octyl chain, whereas the intermediates in the Bloch pathway have unsaturated (single double bond) iso-octyl chain. 7-Dehydrocholesterol (7-DHC) and desmosterol represent immediate biosynthetic precursors of cholesterol in the Kandutsch-Russell and Bloch pathways, respectively. 7-DHC differs from cholesterol *merely* by the presence of an additional double bond at the 7th position in the sterol ring (highlighted in red), whereas desmosterol differs with cholesterol only in a double bond (highlighted in red) at the 24th position in its alkyl side chain. Interestingly, accumulation of either 7-DHC or desmosterol due to defective sterol biosynthesis has been shown to result in serious neurological disorders. The molecular structure of cholesterol has been exceedingly fine-tuned (selected) over billions of years of natural (Darwinian) evolution to support membrane function in higher eukaryotes. This was proposed by Konrad Bloch (the Bloch hypothesis). The Bloch hypothesis assumes relevance in disorders in which a small change in cholesterol structure leads to lethal phenotypes such as the Smith-Lemli-Opitz syndrome (SLOS). The solid and dashed arrows represent single step and multistep reactions, respectively. See text for more details.

Cellular cholesterol biosynthesis takes place predominantly in membranes of the endoplasmic reticulum. Most of the enzymes catalyzing cholesterol biosynthesis, downstream of HMG-CoA reductase (3-hydroxy-3-methylglutaryl coenzyme A reductase), the first rate-limiting enzyme in the biosynthesis pathway, are membrane-bound (Gaylor, 2002). Cholesterol biosynthesis proceeds via two pathways, termed Kandutsch-Russell and Bloch pathways (Kandutsch and Russell, 1960; Bloch, 1965; Nes, 2011; Brown and Sharpe, 2016). The biosynthesis begins with a molecule of acetyl-CoA combining with acetoacetyl-CoA to form HMG-CoA. This is followed by the first rate-limiting step of cholesterol biosynthesis, i.e., conversion of HMG-CoA to mevalonate, catalyzed by HMG-CoA reductase. Mevalonate further undergoes a series of enzymatic reactions to generate the last common substrate for both pathways, i.e., lanosterol (see Figure 1). The only difference between the two pathways lies in the iso-octyl side chain of the intermediates. In the Kandutsch-Russell pathway, the intermediates have a saturated iso-octyl chain, whereas the intermediates in the Bloch pathway have unsaturated iso-octyl chain due to the presence of a double bond (Nes, 2011). Notably, the relative contributions of these two pathways exhibit a marked dependence on age, tissue and cell-type (Mitsche et al., 2015). Interestingly, statins, one of the top selling drugs globally for treatment of hypercholesterolemia and dyslipidemia, are competitive inhibitors of HMG-CoA reductase (Istvan and Deisenhofer, 2001). Statins are structural analogs of HMG-CoA and competitively inhibit the reduction of HMG-CoA to mevalonic acid.

Biosynthesis of cholesterol is an oxygen-intensive process, since synthesis of one molecule of cholesterol requires 11 molecules of oxygen (Brown and Galea, 2010). Lack of cellular oxygen suppresses cholesterol biosynthesis, giving rise to hypoxia (Dutta et al., 2022). The intimate relation between cholesterol biosynthesis and evolution of oxygen was elegantly analyzed by Konrad Bloch (Bloch, 1983; 1994), who was awarded the Nobel Prize in Physiology and Medicine (along with Feodor Lynen) in 1964 for his pioneering work on cholesterol and fatty acid biosynthesis (for a lucid account of Konrad Bloch’s life and science, see Vance and Goldfine, 2002). Konrad Bloch’s work showed that the last linear molecule in the cholesterol biosynthesis pathway, squalene, cannot be cyclized without oxygen. It is even more difficult to carry out further steps in the biosynthesis of cholesterol without oxygen. These steps can be viewed as progressively making the surface of cholesterol less hydrophobic by successive removal of three methyl groups from lanosterol (Mouritsen and Bagatolli, 2016). In fact, the evolution of cholesterol through its biosynthesis pathway could be viewed as an adaptation to increase in atmospheric oxygen level (Bloch, 1994; Brown and Galea, 2010). The oxygen atom in the polar hydroxyl group of cholesterol is introduced in the biosynthesis pathway prior to cyclization by epoxidation of squalene, thereby giving rise to lanosterol, the first cyclic precursor in the cholesterol biosynthetic pathway.

Lanosterol is the last common substrate in the Kandutsch-Russell and Bloch pathways, before the pathway bifurcates. The interplay between cholesterol biosynthesis and natural evolution of cholesterol is elegantly conceptualized in Bloch hypothesis (Bloch, 1983; 1994; Brown and Galea, 2010; Kumar and Chattopadhyay, 2016). Close to four decades back, Konrad Bloch speculated that the cholesterol biosynthetic pathway parallels cholesterol evolution. According to this hypothesis, the molecular structure of cholesterol has been fine-tuned (i.e., selected, in an evolutionary perspective) over a very long timescale of natural (Darwinian) evolution (∼billions of years) for its ability to optimize certain physical properties of eukaryotic cell membranes in the context of membrane function (Mouritsen and Bagatolli, 2016). Cholesterol precursors should therefore have properties that gradually support cell membrane function in higher organisms as they progress along the pathway toward cholesterol. In other words, the order of synthesis of sterols in the cholesterol biosynthesis pathway is indicative of their distance in evolutionary time, with evolution perfecting the molecule for optimal membrane function in higher eukaryotes (Konrad Bloch termed it as “evolutionary perfection of a small molecule” (Bloch, 1994; also see Galea and Brown, 2009; Stevenson and Brown, 2009). Essentially this means that each subsequent intermediate in the cholesterol biosynthesis pathway is superior in supporting membrane function in higher eukaryotes relative to its precursor. *This is validated by metabolic disorders wherein any change in the cholesterol biosynthetic pathway (due to mutations in the enzymes involved in the pathway) could lead to accumulation of cholesterol precursors resulting in lethal phenotypes (for example, see discussion below on SLOS)*.

## Smith-Lemli-Opitz syndrome: Molecular etiology and treatment

**FIGURE 2:**
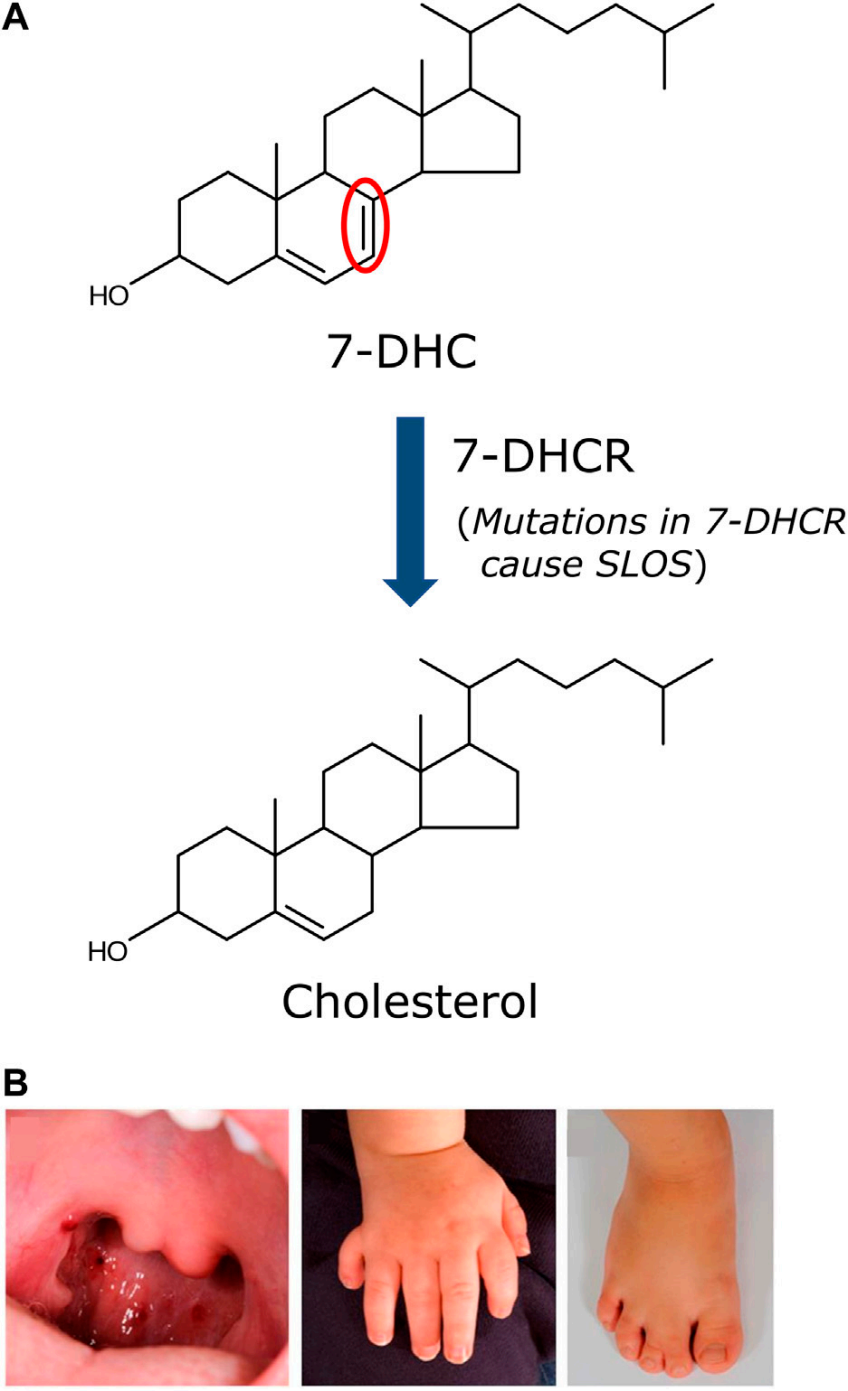
Biochemical and physical defects associated with SLOS. **(A)** The etiology of SLOS lies in the defective last step in the Kandutsch-Russell pathway of cholesterol biosynthesis, i.e., the conversion of 7-DHC to cholesterol. The enzyme catalyzing this step (7-DHCR) loses its ability to efficiently convert 7-DHC to cholesterol due to mutations in 7-DHCR detected in SLOS patients. More than 150 mutations in 7-DHCR have been reported in SLOS patients. This results in accumulation of 7-DHC and an altered cholesterol/total sterol ratio, a clinical parameter used to detect and diagnose SLOS. The severity of SLOS depends on the degree of functional impairment of the enzyme. The only difference between cholesterol and 7-DHC is the mere presence of an additional double bond (highlighted in red). **(B)** Representative anatomical defects displayed by SLOS patients. The anatomical defects associated with SLOS include cleft palate, polydactyly and syndactyly. Adapted and modified from (Porter and Herman 2011).

The Smith-Lemli-Opitz syndrome (SLOS) is a congenital error of cholesterol biosynthesis, resulting in an autosomal recessive disorder, which leads to developmental, behavioral and cognitive abnormalities (Waterham and Wanders, 2000; Chattopadhyay and Paila, 2007; Porter, 2008; Thurm et al., 2016). SLOS was first reported by three physicians (David Smith, Luc Lemli and John Opitz) almost 60 years back (Smith et al., 1964). However, the unraveling of the molecular mechanism underlying the disease had to wait for three more decades when it was reported that the etiology of SLOS lies in defective cholesterol biosynthesis, giving rise to low plasma cholesterol concentration accompanied with accumulation of a cholesterol precursor, 7-dehydrocholesterol (7-DHC), in patients (Irons et al., 1993; Tint et al., 1994). In the next few years, a number of groups identified mutations in the gene encoding the enzyme 7-dehydrocholesterol reductase (7-DHCR) in the last step of the Kandutsch-Russell pathway of cholesterol biosynthesis (see Figure 1) (Fitzky et al., 1998; Wassif et al., 1998; Waterham et al., 1998) and the DHCR7 gene was cloned (Moebius et al., 1998). The enzyme 7-DHCR reduces the Δ^7^ double bond in 7-DHC to form cholesterol (Figure 2A). Mutations in 7-DHCR lead to accumulation of 7-DHC and its positional isomer 8-DHC and subsequent changes in cholesterol/total sterol ratio (Batta et al., 1995; Griffiths et al., 2017). More than 150 mutations have been reported to cause SLOS (Waterham and Hennekam, 2012). The severity of the syndrome depends on the degree of functional impairment in the enzyme 7-DHCR. Based on the severity, SLOS is divided into two types: a less severe Type I and a more severe Type II (Tint et al., 1995a) syndrome. Patients typically live from a few days to up to 30 years or more. However, they suffer from intellectual disability, behavioral problems and anatomical defects (malformations). Patients diagnosed with SLOS typically show slow growth and intellectual disability, microcephaly, cleft palate, syndactyly, polydactyly, urogenital abnormalities, and a variety of other anatomical defects (Figure 2B) (Porter and Herman, 2011). Learning disabilities and intellectual disability are present in at least 95% of SLOS patients (Porter, 2000) which could be attributed to impaired neurotransmission.

The typical carrier frequency for SLOS is reported to be greater in Caucasian population with incidence of 1:10,000 to 1:70,000 and a carrier frequency of 1:30 (Battaile et al., 2001; Porter and Herman, 2011). The relationship between clinical severity and biochemical parameters in SLOS continue to be an ongoing area of research and lacks consensus. It has been earlier reported that reduced cholesterol levels are associated with increased clinical severity, whereas the correlation between 7-DHC levels and clinical severity is weak (Cunniff et al., 1997). These results are supported using a SLOS rat model, which shows that cholesterol deficit but not accumulation of precursor sterols, is the major cause of abnormal embryogenesis in SLOS (Gaoua et al., 2000). On the other hand, only a modest correlation (∼40%) was found between severity and plasma cholesterol in SLOS patients (Yu et al., 2000). Interestingly, the ratio of 7-DHC (and 8-DHC) to cholesterol has been reported to be linearly related to impairment in cognitive and adaptive function, with the amount of 7-DHC accumulation being the most important determinant (Thurm et al., 2016). This adds to the ongoing discussion on whether it is the depletion of cholesterol or accumulation of 7-DHC (or the oxysterols derived from 7-DHC), which causes SLOS. Since SLOS is characterized by reduced levels of plasma cholesterol along with accumulation of 7-DHC and its positional isomer 8-DHC (Porter, 2008), clinical characterization of SLOS is predominantly carried out by measurement of altered 7-DHC (and 8-DHC)/cholesterol ratio in the blood serum (Tint et al., 1995b). However, there could be additional factors involved in the pathology of SLOS such as oxysterols and hydroxyl derivatives of 7- and 8-DHC, which are suggested to complement defects associated with SLOS (Wassif et al., 2003; Korade et al., 2010; Xu et al., 2011; Xu et al., 2012; Griffiths et al., 2017; Li et al., 2022; Tomita et al., 2022). A recent report has shown that one of the oxidative derivatives of 7-DHC, i.e., 3β, 5α-dihydroxycholest-7-en-6-one (DHCEO) is involved in neurogenic defects in SLOS by causing aberrant premature neurogenesis in both mouse and neural progenitor cells, which could be rescued with antioxidants (Tomita et al., 2022).

At present, there is no specific drug available to treat SLOS. A defect in the cholesterol biosynthesis pathway led to the obvious recommendation of dietary cholesterol supplementation as the first therapy to treat SLOS patients (Elias et al., 1997). In addition, use of statins (simvastatin, in particular) along with dietary cholesterol has been suggested (Chan et al., 2009). The choice of simvastatin is based on it being one of the most lipophilic of all available statins, and therefore, the one most likely to cross the blood-brain-barrier to exert its desired effects in the brain (Jira et al., 2000). Simvastatin is a semi-synthetic, highly lipophilic statin (Serajuddin et al., 1991), and is believed to be one of the most effective statins (Schaefer et al., 2004). Earlier studies reported that simvastatin has a high octanol-water partition coefficient relative to other hydrophilic statins (Serajuddin et al., 1991) that allows it to partition into model membranes (Galiullina et al., 2017; Sahu et al., 2019; Sariisik et al., 2019) and cross the blood-brain-barrier (Saheki et al., 1994; Wood et al., 2010; Sierra et al., 2011). Although it may appear counterintuitive to use a cholesterol lowering drug such as simvastatin in treatment of SLOS, which is characterized by cholesterol deficiency, it is hoped that by blocking the cholesterol biosynthesis pathway proximal to the location of the defect in SLOS, the abnormally high levels of potentially toxic 7-DHC (and 8-DHC) could be reduced (Svoboda et al., 2012). Further, to reduce the effects caused due to derivatives of 7-DHC and 8-DHC, antioxidants have been recommended (Fliesler, 2013). However, none of the therapies recommended by clinicians have gone through the rigor of a comprehensive trial (Svoboda et al., 2012).

## Defective Hedgehog signaling in SLOS

The developmental abnormalities observed in SLOS patients are believed to be due to defective signaling of Sonic Hedgehog, the protein implicated in development and pattern formation (Mullor et al., 2002; Cooper et al., 2003; Koide et al., 2006; Blassberg and Jacob, 2017). SLOS is a developmental disorder wherein changes in cellular sterol levels affect various stages of development which in turn cause anatomical and behavioral defects. Some of the abnormal morphological features reported in SLOS (such as holoprosencephaly, an abnormality where forebrain fails to separate into right and left hemispheres) are also observed upon mutations in components of the Sonic Hedgehog signaling pathway (Kelly et al., 1996; Roessler et al., 1996). The hedgehog signaling pathway is an important pathway involved in development, growth, and homeostasis. A defect in hedgehog signaling is associated with several developmental defects and tumors (Rubin and de Sauvage, 2006; Briscoe and Thérond, 2013). The role of membrane lipids in regulating hedgehog pathway is an ongoing area of research and the precise role of lipids in this process is not well established yet.

**FIGURE 3:**
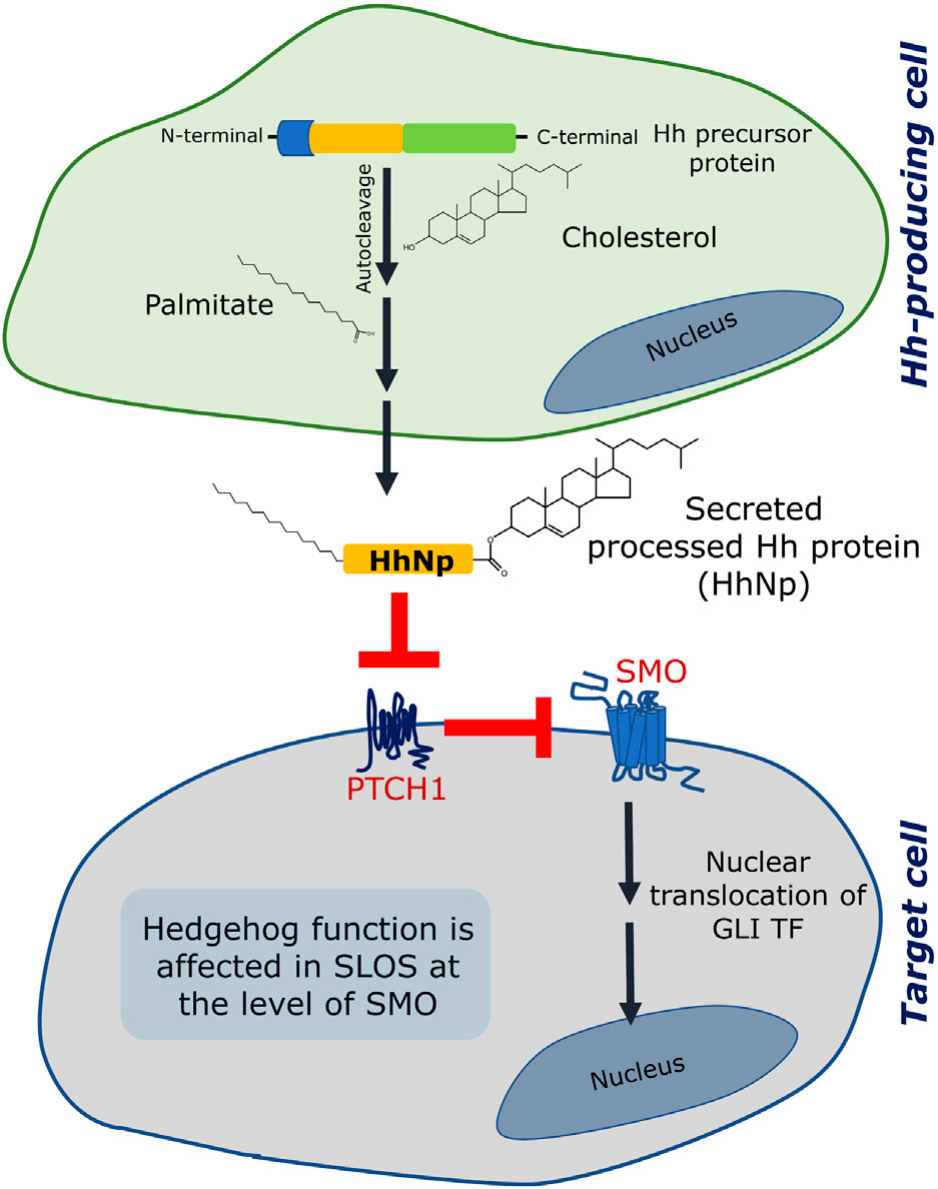
Hedgehog (Hh) signaling is affected in SLOS. Hedgehog signaling involves two sites: (i) a Hedgehog producing cell and (ii) a responding/target cell. The Hedgehog protein is formed as a precursor protein in the generating cell, which is processed by an autocleavage reaction followed by two lipid modification reactions, one of which is esterification of cholesterol to the C terminal of the processed ligand (the other one is palmitoylation). Once fully processed, the Hh ligand (designated as HhNp) is secreted to the target cell. In a target cell, two transmembrane proteins (Patched 1 (PTCH1) and Smoothened (SMO)) act consecutively in response to the Hh (HhNp) ligand. In absence of the ligand, PTCH1 inhibits SMO and therefore inhibits signaling. The Hh ligand binds and inhibits PTCH1 which leads to activation of SMO and subsequent signaling terminating in activation and nuclear translocation of GLI transcription factors (TF). Hedgehog signaling is affected in SLOS at the level of SMO, which could be due to inefficiency of 7-DHC to activate SMO or due to lack of cholesterol. See text for more details.

Hedgehog signaling involves a sequence of events which results in activation of downstream signaling. The ligands (termed the Hedgehog ligands or Hh ligands) are processed in a Hh-producing cell and secreted toward distant target cells. The processing of a Hh ligand includes an autocatalytic cleavage which results into a lipid-modified amino-terminal fragment which acts as a ligand for activating signaling in the target cell (Porter et al., 1996; Eaton, 2008) (see Figure 3). The lipid modifications are a) a covalent linkage of a cholesterol molecule to the carboxyl terminus of the N-terminal fragment and b) palmitoylation on a cysteine residue near its N terminus. The processed Hh ligands are secreted as multimeric complexes by Dispatched, which then travel to the target cell and activate signaling. In the target cell, the first player is Patched 1 (PTCH1) which inhibits Smoothened (SMO, a GPCR) in the absence of Hh ligand. Activation of PTCH1 by Shh relieves SMO which becomes active and induces downstream signaling which culminates into nuclear translocation of GLI transcription factors. It has been reported that cholesterol is an important regulator of Hedgehog signaling at various stages such as in the processing of Hh ligand, its assembly into multimeric complexes, its cellular localization and trafficking, release from the cells, distant signaling and stabilization in extracellular matrix (Gallet et al., 2003; Callejo et al., 2006; Gallet et al., 2006; Li et al., 2006). In addition, cholesterol affects Hedgehog signaling at the level of target cell interacting partners such as SMO.

Since SLOS is a developmental disorder associated with defective cholesterol metabolism, it is not surprising to have defective Hedgehog signaling considering Hedgehog’s crucial role in development. As mentioned above, patients suffering from SLOS display developmental malformations consistent with defects reported upon abnormal Hedgehog signaling (Roessler et al., 1996). Notably, rats treated with potent inhibitors of 7-DHCR such as AY 9944 or BM 15.766 show similar developmental defects (Cooper et al., 1998). One of the unresolved questions in SLOS is whether the symptoms observed could arise due to reduction in cholesterol or elevation of 7-DHC (and its derivatives including oxysterols) levels or a combination of both. In case of Hedgehog signaling, some of these questions have been debated. In the first step of Hedgehog signaling pathway, *i.e.,* autoprocessing of the Hh ligand, it has been reported that inhibition of cholesterol biosynthetic pathway using AY 9944 in cultured cells did not affect the autocatalytic cleavage of the Shh ligand and the sterol modification of the ligand (Cooper et al., 1998). However, signaling was reported to be inhibited upon AY 9944 treatment in a dose-dependent manner. In another study, autoprocessing of the Shh ligand in cultured Chinese hamster ovary (CHO) cells was completely blocked in severe acute cholesterol depletion conditions generated using the cyclic oligosaccharide methyl-β-cyclodextrin (MβCD) (Guy, 2000). However, it was reported that when embryonic fibroblasts from mouse model of SLOS were cultured, Shh processed normally, whereas the cells were unable to respond to the ligand when transiently treated with MβCD and cultured in delipidated medium (Cooper et al., 2003). Importantly, SMO has been suggested to be the site of sterol action in these studies. This was supported by another report in which reduced levels of cholesterol were suggested to be the cause of defective SMO activation in fibroblasts obtained from a mouse model of SLOS (Blassberg et al., 2016). In addition, it has been reported that DHCEO blocks activation of the Hedgehog pathway at the level of SMO which could be detrimental to embryonic development (Sever et al., 2016). In a recent study, it has been reported that acute increase in plasma membrane cholesterol is sufficient to induce Hedgehog signaling and cholesterol could enhance signaling strength mediated by the native ligand (Luchetti et al., 2016).

Taken together, it appears that Hedgehog signaling is affected in SLOS at the level of SMO. SMO is a member of the GPCR family of receptors, one of the most studied classes of membrane receptors involved in signal transduction across the plasma membrane (Katritch et al., 2013; Chattopadhyay, 2014; Sakmar, 2017). Although the role of cholesterol in mediating a varied aspects of GPCR function such as signaling, stability, oligomerization, endocytosis and trafficking constitutes an exciting contemporary area of research (Pucadyil and Chattopadhyay, 2006; Paila and Chattopadhyay, 2010; Oates and Watts, 2011; Goddard and Watts, 2012; Chattopadhyay, 2014; Sengupta and Chattopadhyay, 2015; Gimpl, 2016; Sengupta et al., 2018; Jafurulla et al., 2019; Kiriakidi et al., 2019; Sarkar et al., 2020; Jakubík and El-Fakahany, 2021; Kumar and Chattopadhyay, 2021a; Sarkar et al., 2022; Sarkar and Chattopadhyay, 2022), activation of SMO by cholesterol has recently added a new functional dimension to this area of work (Luchetti et al., 2016; Kinnebrew et al., 2022; Kumari et al., 2022).

## Can 7-DHC substitute for cholesterol in neurotransmitter receptor function? The case of the serotonin_1A_ receptor

Since SLOS is associated with anatomical deformities and neurological dysfunction, exploring the function of neuronal receptors and their interaction with membrane cholesterol assumes significance. In this context, the serotonin_1A_ receptor is an important and relevant neurotransmitter receptor belonging to the GPCR superfamily of receptors (Pucadyil et al., 2005; Kalipatnapu and Chattopadhyay, 2007; Müller et al., 2007; Sarkar et al., 2018; Sarkar et al., 2021). The serotonin_1A_ receptor modulates many neurological functions and is a major drug target for treating psychiatric disorders such as anxiety, stress, depression, learning deficiency, cognition, schizophrenia and Parkinson’s disease (Lacivita et al., 2008; Akimova et al., 2009; Ohno, 2011; Celada et al., 2013; Fiorino et al., 2014; Kaufman et al., 2016; Carhart-Harris and Nutt, 2017). Previous work from our laboratory has extensively reported the crucial role of membrane cholesterol in organization, dynamics, endocytosis and function of the serotonin_1A_ receptor (Pucadyil and Chattopadhyay, 2006; Paila and Chattopadhyay, 2010; Jafurulla and Chattopadhyay, 2013; Jafurulla et al., 2014; Sengupta and Chattopadhyay, 2015; Jafurulla et al., 2019; Kumar and Chattopadhyay, 2020; Sarkar et al., 2020; Kumar and Chattopadhyay, 2021b).

**FIGURE 4:**
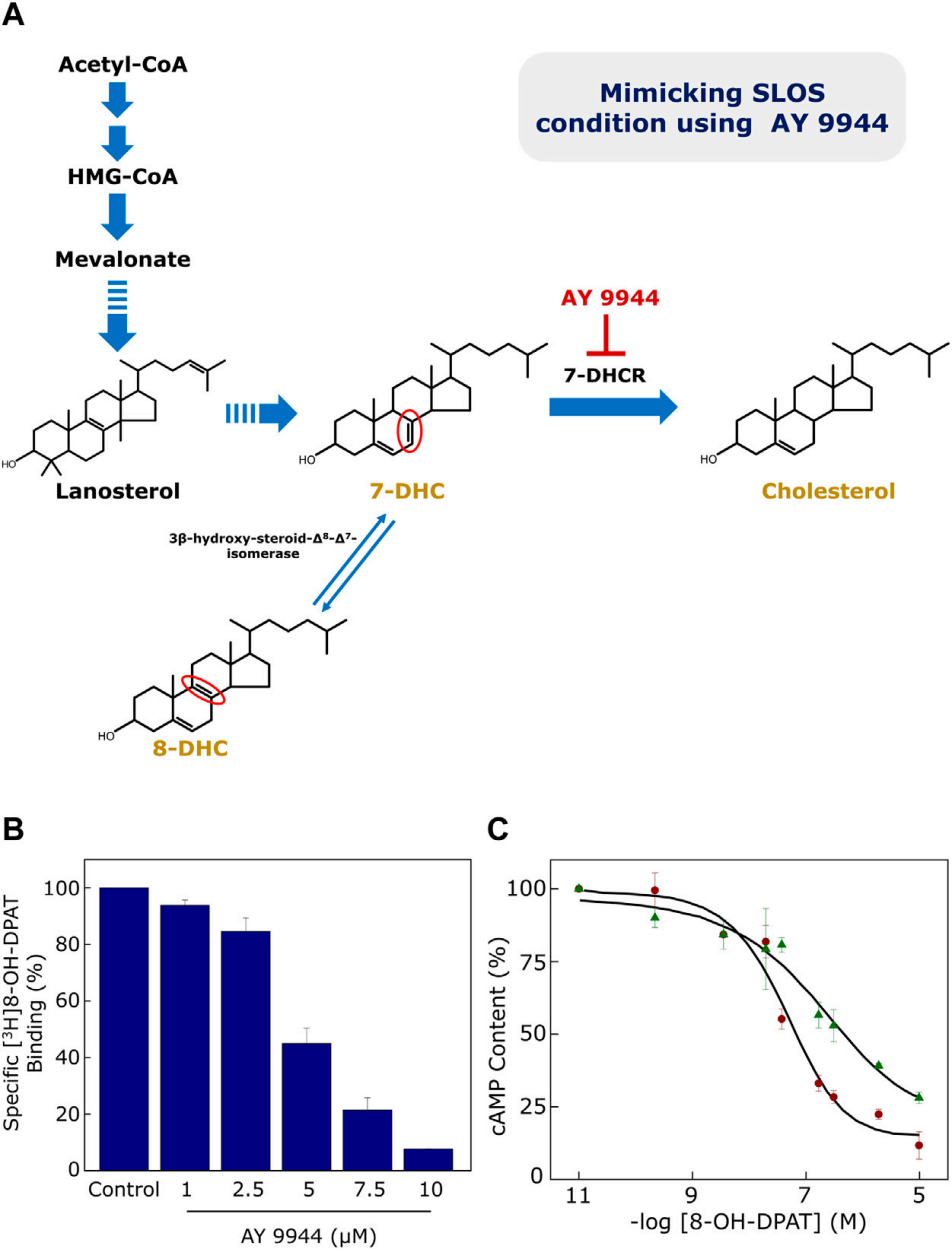
**(A)** Generation of a cellular model of SLOS using AY 9944, a specific metabolic inhibitor of 7-DHCR, the enzyme that reduces the Δ^7^ double bond in 7-DHC in the last step of the Kandutsch-Russell pathway of cholesterol biosynthesis (see Figures 1, 2). Mutations in 7-DHCR cause SLOS. Reduced levels of cholesterol, accompanied with elevated levels of dehydrocholesterol (7-DHC + 8-DHC), are characteristic of SLOS. 8-DHC, a positional isomer of 7-DHC, is formed due to an enzyme-catalyzed isomerization of 7-DHC. The only difference between 7-DHC (and 8-DHC) and cholesterol is the double bond at the 7th (or 8th) position in the sterol ring (highlighted in their chemical structures in red). Ligand binding and cellular signaling of the serotonin_1A_ receptor is affected in the cellular model of SLOS (see (B) and (C) below). **(B)** CHO cells stably expressing the serotonin_1A_ receptor were treated with increasing concentrations of AY 9944 and specific [^3^H]8-OH-DPAT binding was measured. Ligand binding activity of the serotonin_1A_ receptor exhibited a progressive reduction with increasing concentrations of AY 9944. **(C)** Downstream signaling in the cellular model of SLOS was determined by measuring cellular cAMP levels. The serotonin_1A_ receptor is negatively coupled to G-proteins (Gi) and therefore leads to reduction in cellular cAMP level upon stimulation with agonists. Data represent changes in cellular cAMP level in control (maroon circles) cells and cells treated with 5 μM AY 9944 (green triangles) with increasing concentrations of 8-OH-DPAT. Adapted and modified from Paila et al. (2008) with permission from Elsevier. See text for more details.

Since SLOS is a disease characterized by low plasma cholesterol, we previously generated a cellular model of SLOS using CHO cells stably expressing the human serotonin_1A_ receptor (Paila et al., 2008). For this, we employed the strategy of inhibiting cholesterol biosynthesis by using a specific inhibitor (AY 9944) of 7-DHCR (Dvornik et al., 1963) in the final step of the Kandutsch-Russell pathway of cholesterol biosynthesis (see Figures 1, 2, 4A). To monitor the function of the human serotonin_1A_ receptor in SLOS-like condition, we utilized this cellular model. We observed depletion of cholesterol and accumulation of 7-DHC and its positional isomer 8-DHC in the cellular model. Notably, the ratio of these sterols matched the ratios reported in SLOS patients. To explore the effect of SLOS-like condition on the serotonin_1A_ receptor function, we examined the functional aspects of the serotonin_1A_ receptor at various stages of signaling, i.e., ligand binding, G-protein coupling and intracellular signaling. We observed that ligand binding (Figure 4B) and downstream signaling (Figure 4C) of the serotonin_1A_ receptor are impaired in SLOS-like condition (Paila et al., 2008). Upon metabolic replenishment of cholesterol (by using serum), we could partially restore the ligand binding activity of the serotonin_1A_ receptor. We further showed that acute depletion of cholesterol followed by replenishment with 7-DHC could not restore ligand binding of the serotonin_1A_ receptor in hippocampal membranes, thereby highlighting the intrinsic structural stringency necessary for interaction of cholesterol with the serotonin_1A_ receptor (Singh et al., 2007). Taken together, these results show that 7-DHC could not substitute for cholesterol in maintaining the normal function of the serotonin_1A_ receptor.

## Endocytosis and cellular trafficking: New paradigm in SLOS research?

Mammalian cells have evolved a variety of mechanisms to internalize molecules of various sizes and particles, collectively termed endocytosis (Mukherjee et al., 1997). Endocytosis is a key mechanism by which cargoes are delivered from outside the cell to the cellular interior. It ranges from a simple uptake of fluid from cell surroundings (pinocytosis) to uptake of particles such as bacteria and cellular debris (phagocytosis) to a more selective uptake of specific molecules via receptors (receptor-mediated endocytosis) (Doherty and McMahon, 2009).

Endocytosis involves a complex interplay between membrane-associated and cytoplasmic proteins, which contributes to recruitment of cargo, generation of local membrane curvature and budding of cargo-laden vesicles from the plasma membrane. However, the role of membrane lipids in the process of endocytosis is relatively less explored. It has been previously reported that acute depletion of cholesterol with MβCD resulted in reduction in the rate of internalization of the transferrin receptor accompanied with accumulation of flat clathrin-coated membranes and reduction in deep clathrin-coated pits (Subtil et al., 1999). In another work, acute depletion of cholesterol with MβCD was reported to inhibit the endocytosis of transferrin and epidermal growth factor, that could be reversed upon replenishment of cholesterol (Rodal et al., 1999). Taken together, these reports show that clathrin-mediated endocytosis is sensitive to acute cholesterol depletion due to the inability of clathrin-coated membrane regions to pinch off to form coated vesicles. Interestingly, in case of the nicotinic acetylcholine receptor, an ion channel, acute cholesterol depletion by MβCD was reported to enhance the kinetics of internalization of the receptor (Borroni et al., 2007; Borroni and Barrantes, 2011). In case of pharmacologically relevant GPCRs, early reports showed that acute cholesterol depletion affects the endocytosis of δ-opioid receptor 1 (Brejchova et al., 2016), LPA_1_ lysophosphatidic acid receptor (Urs et al., 2005) and melanocortin-4 receptor (McDaniel et al., 2012). Recent results from our laboratory have revealed that the endocytosis and trafficking of serotonin_1A_ receptor (a neurotransmitter receptor) could be modulated by cholesterol (Kumar and Chattopadhyay, 2020; 2021a; b; Sharma et al., 2021). Notably, the actual mode of cholesterol depletion (acute vs. chronic) appears to be crucial in determining the fate of the receptor. Acute cholesterol depletion by MβCD led to inhibition of endocytosis of the serotonin_1A_ receptor (Kumar and Chattopadhyay 2021b). In contrast, chronic metabolic depletion of cholesterol by lovastatin resulted in a switch in the mechanism of endocytosis from clathrin- to caveolin-mediated endocytosis, along with re-routing of intracellular traffic to lysosomes instead of the plasma membrane (Kumar and Chattopadhyay 2020). These results highlight the complexity involved in these two processes and emphasize the relevance of the actual methodology used to deplete cholesterol (for more details, see Sarkar et al., 2017; Kumar and Chattopadhyay, 2021a). In addition, we showed that inhibition of sphingolipid biosynthesis could affect trafficking of the serotonin_1A_ receptor (Kumar et al., 2021).

In this overall context, and keeping in mind that SLOS is a disorder characterized by imbalance of cholesterol due to defective biosynthesis (low cholesterol in patient serum), endocytosis and trafficking of relevant neurotransmitter receptors assume significance. Notably, in a recent work, it has been shown that the clathrin-mediated endocytosis of transferrin receptors was significantly reduced in fibroblasts from SLOS patients (Anderson et al., 2021). Importantly, the extent of inhibition of clathrin-mediated endocytosis correlated well with the total sterol content in SLOS cell lines derived from patient fibroblasts. This was attributed to the ability of cholesterol to be able to bend membranes by lowering energy barriers of membrane bending which helps in clathrin-mediated endocytosis. In addition, clathrin-mediated endocytosis could not be fully restored in cells exhibiting severe SLOS phenotype upon supplementation with serum containing lipoproteins. In the same study, it was reported that mimicking SLOS conditions *in vitro* using AY 9944 resulted in reduction in transferrin receptor endocytosis, similar to what was observed in patient samples. Additionally, replenishment with two sterols, i.e., cholesterol and 7-DHC could rescue the internalization of the receptor. Interestingly, defective folate uptake (via folate receptor internalization) has been reported in fibroblasts obtained from SLOS patients (Tulenko et al., 2006). Folate deficiency affects palate formation and therefore, could be a possible reason for observed cleft palate in SLOS patients (Wehby and Murray, 2010).

In addition to acting as a mechanism to uptake cargoes from cellular surroundings, endocytosis of GPCRs serve as an important regulatory mechanism to sustain downstream signaling by GPCRs within a stringent spatiotemporal regime. Endocytosis of GPCRs is one of the most studied regulatory mechanisms which helps in spatially decoupling a part of plasma membrane receptors from its pool of extracellular ligands by compartmentalizing GPCRs in intracellular vesicles (endosomes) (Ferguson, 2001; Hanyaloglu and von Zastrow, 2008). Endocytosis therefore stringently restricts the number of plasma membrane receptors available for signaling. Interestingly, there have been several reports suggesting that the internalized receptors also mediate signaling and the signaling mediated by the internalized GPCR could be different from that on the plasma membrane (Irannejad et al., 2013; Irannejad et al., 2015; Lohse and Hofmann, 2015; Bowman et al., 2016; Eichel and von Zastrow, 2018; Jong et al., 2018; Thomsen et al., 2018; Lobingier and von Zastrow, 2019; Crilly et al., 2021; Crilly and Puthenveedu, 2021; Kumar and Puthenveedu, 2022).

The role of membrane lipids in regulating the localization of GPCRs is an emerging area of interest. The first conceptual understanding of lipid-defined cellular localization was proposed by Mark Bretscher and Sean Munro when they proposed that increase in cholesterol content from ER to Golgi to plasma membrane could help in sorting of membrane proteins along the secretory pathway (Bretscher and Munro, 1993). The rationale behind this was hypothesized to be based on progressive thickening of membranes (due to increased cholesterol concentration) along the biosynthetic machinery (ER-Golgi-plasma membrane), so that the plasma membrane is the thickest and ER the thinnest membrane. Membrane proteins have transmembrane regions of different lengths based on their intracellular localization, depending on membrane thickness of the organelle where they are localized (Sharpe et al., 2010). This essentially means that a change in membrane physical properties (such as thickness) could affect localization of membrane proteins (due to hydrophobic mismatch (Rao et al., 2017)).

**FIGURE 5:**
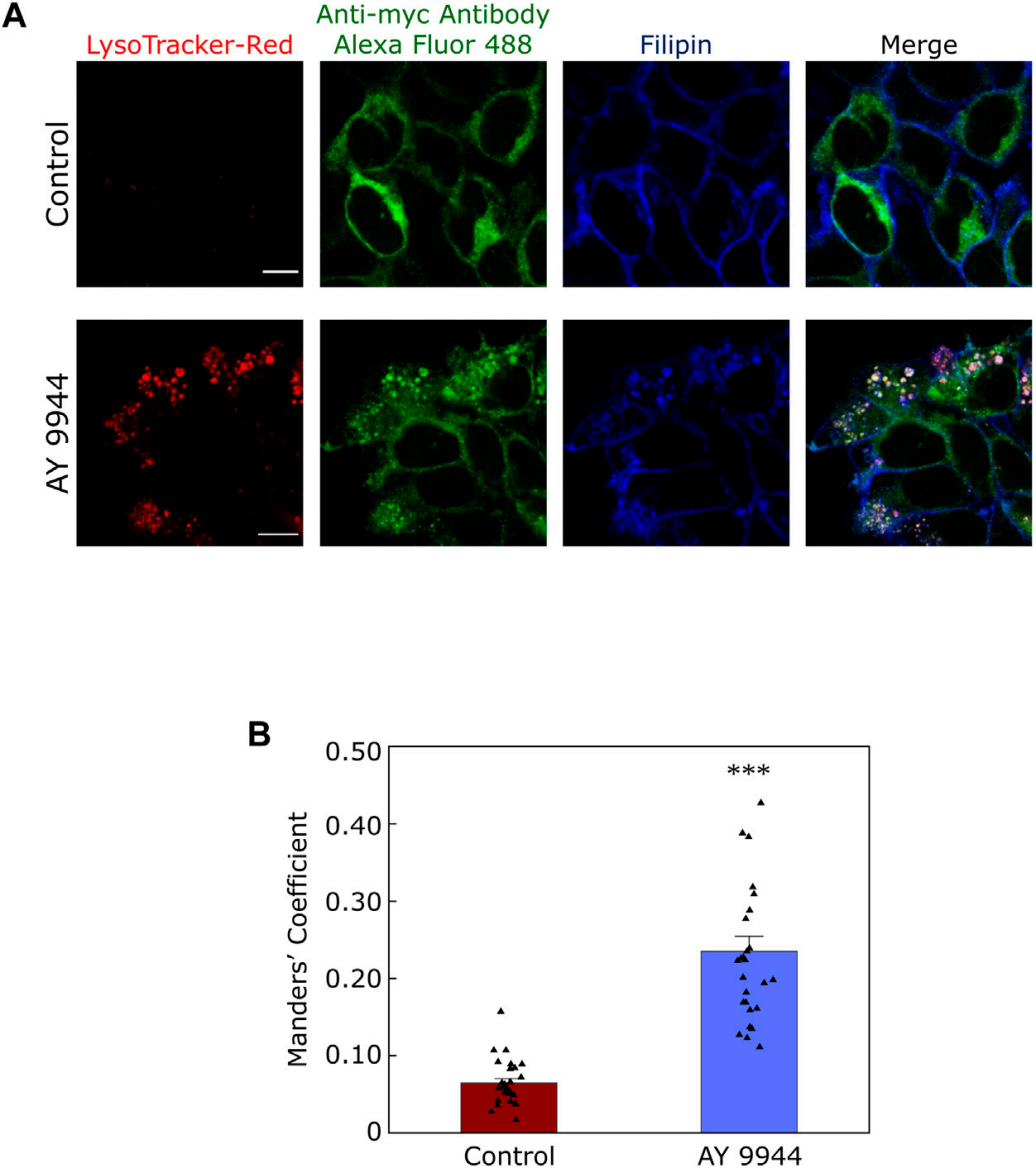
Defective trafficking of a neurotransmitter receptor in a cellular model of SLOS. Serotonin_1A_ receptors are predominantly localized in the plasma membrane in HEK-293 cells stably expressing human serotonin_1A_ receptors myc-tagged at the N-terminal (HEK-5-HT_1A_R cells). We generated a cellular model of SLOS by chronic treatment of HEK-5-HT_1A_R cells with AY 9944, a specific inhibitor of 7-DHCR, the enzyme that converts 7-DHC to cholesterol in the last step of the Kandutsch-Russell pathway of cholesterol biosynthesis (see Figures 1, 4). Our results show a considerable reduction in the plasma membrane population of the serotonin_1A_ receptor in SLOS-mimicking condition. In addition, we observed accumulation of sterol-enriched LysoTracker positive compartments in these conditions (see LysoTracker-Red mid plane sections in control and AY 9944 treated conditions). The loss in plasma membrane localization was accompanied by an increased localization of the serotonin_1A_ receptor in sterol-enriched LysoTracker positive compartments, indicating that there is altered trafficking of serotonin_1A_ receptors in SLOS-like condition. **(A)** The panel shows representative confocal microscopic images of the mid-plane section showing the extent of colocalization between LysoTracker-Red (red), the serotonin_1A_ receptor (green) and filipin (blue) in control (untreated) conditions and those treated with AY 9944 [the extent of colocalization is shown by Manders’ colocalization coefficient, see panel **(B)**]. Scale bars represent 10 μm. Serotonin_1A_ receptors were tagged with anti-myc antibody Alexa Fluor 488 conjugate. Filipin is a polyene antibiotic which labels free (unesterified) sterols. Late endosomal/lysosomal compartments were labeled with LysoTracker-Red. **(B)** Quantitative estimates of the extent of colocalization of the serotonin_1A_ receptor with LysoTracker-Red in control (maroon) and AY 9944 treated cells (blue) expressed as Manders’ coefficient. The concentration of AY 9944 was 2 μM in all cases. Adapted and modified with permission from Sharma et al., 2021. See text for more details.

In this context, our recent results showing a reduction in plasma membrane population of the serotonin_1A_ receptors under SLOS-mimicking condition and intracellular accumulation of the receptor in sterol-enriched late endosomal/lysosomal compartments (Sharma et al., 2021) assume relevance. We created a cellular model of SLOS in HEK-293 cells stably expressing the human serotonin_1A_ receptor (HEK-5-HT_1A_R cells) using AY 9944. This resulted in accumulation of 7-DHC in these cells along with an altered cholesterol/total sterol ratio, similar to earlier reports. In our previous work, we reported that the serotonin_1A_ receptor localizes on the plasma membrane in HEK-5-HT_1A_R cells (Kumar et al., 2019). In the cellular model of SLOS, we observed that there was a significant decrease in the plasma membrane population of the serotonin_1A_ receptor. Western blot analysis showed that this reduction in the plasma membrane population of the receptor was not due to decrease in the overall expression of the receptor. Upon further probing, we observed a considerable increase in sterol-enriched LysoTracker positive compartments, with accumulation of serotonin_1A_ receptors in these compartments (see Figure 5). These results indicate altered trafficking of the serotonin_1A_ receptors in SLOS. To the best of our knowledge, this is the first report in which altered trafficking of a neurotransmitter GPCR has been reported in SLOS-like condition. Notably, accumulation of lysosomal inclusion bodies has been earlier reported in fibroblasts obtained from SLOS patients (Wassif et al., 2002).

The altered location of the serotonin_1A_ receptor in SLOS-mimicking condition could have its origin in differential physicochemical properties of 7-DHC relative to cholesterol. Although 7-DHC differs from cholesterol *merely* by the presence of an additional double bond at the 7^th^ position in the sterol ring (see Figure 2A), biophysical studies show that the presence of 7-DHC could alter membrane physical properties in a significant way. A hallmark of cholesterol is its well documented property of increasing membrane thickness (Nezil and Bloom, 1992) which could modulate lipid-protein interactions in the secretory pathway (Bretscher and Munro, 1993; Sharpe et al., 2010). In spite of the subtle difference in its structure with cholesterol, 7-DHC has been reported to be less effective (relative to cholesterol) in increasing membrane thickness (Rόg et al., 2008; Ren et al., 2011). Membranes containing 7-DHC have been reported to exhibit greater sterol tilt angle (Rόg et al., 2008) which could influence membrane thickness. In addition, 7-DHC has been reported to differ with cholesterol in several membrane properties such as membrane packing (Berring et al., 2005; Shrivastava et al., 2008), phase behavior (Wolf and Chachaty, 2000; Staneva et al., 2010), interfacial temporal heterogeneity (Shrivastava et al., 2020) and membrane dipole potential (Haldar et al., 2012; Singh et al., 2013). This is well supported by an earlier report (Tulenko et al., 2006) in which it was reported that membranes prepared from skin fibroblasts of SLOS patients exhibited abnormal electron density (indicating atypical membrane organization), resulting in reduced molecular packing and increased membrane fluidity. Notably, model membranes mimicking SLOS membranes have been reported to exhibit altered curvature (Gondré-Lewis et al., 2006). In addition, it has been reported that 7-DHC could contribute to destabilizing membrane “rafts” in SLOS (Keller et al., 2004; Kovarova et al., 2006; Megha et al., 2006; Valencia et al., 2006; Staneva et al., 2016).

## Defective autophagy in SLOS

Autophagy is a conserved cellular degradation mechanism to degrade cellular components which are either not required or which have lost their functionality (Glick et al., 2010; Mizushima and Komatsu, 2011). The components degraded via autophagy include proteins, nucleotides, lipids and cellular organelles. The components to be degraded are enclosed in autophagosomes, which then fuses with lysosomes (Lőrincz and Juhász, 2020). Lysosomal degradation helps to break these components and finally recycle back for use in cellular metabolism. In a previous study, it was reported that autophagosome levels increase in SLOS fibroblasts and cells treated with AY 9944, measured by enhanced levels of autophagic markers LC3B-II and beclin-1 (Chang et al., 2014; Rao et al., 2018). Importantly, the levels of LC3B-II showed a positive correlation with cellular concentration of 7-DHC, which highlights the role of cellular sterols in regulating autophagy. Since autophagosomes deliver their cargo to lysosomes, it is possible that accumulation of lysosomal compartments (as reported by us in SLOS-mimicking conditions (Sharma et al., 2021; see above) could provide another feedback mechanism to regulate autophagosome levels in SLOS cells since defective lysosomal function has been reported to induce autophagy (Li et al., 2013).

## Is SLOS a trafficking defect disorder? New therapies based on trafficking defects

**FIGURE 6:**
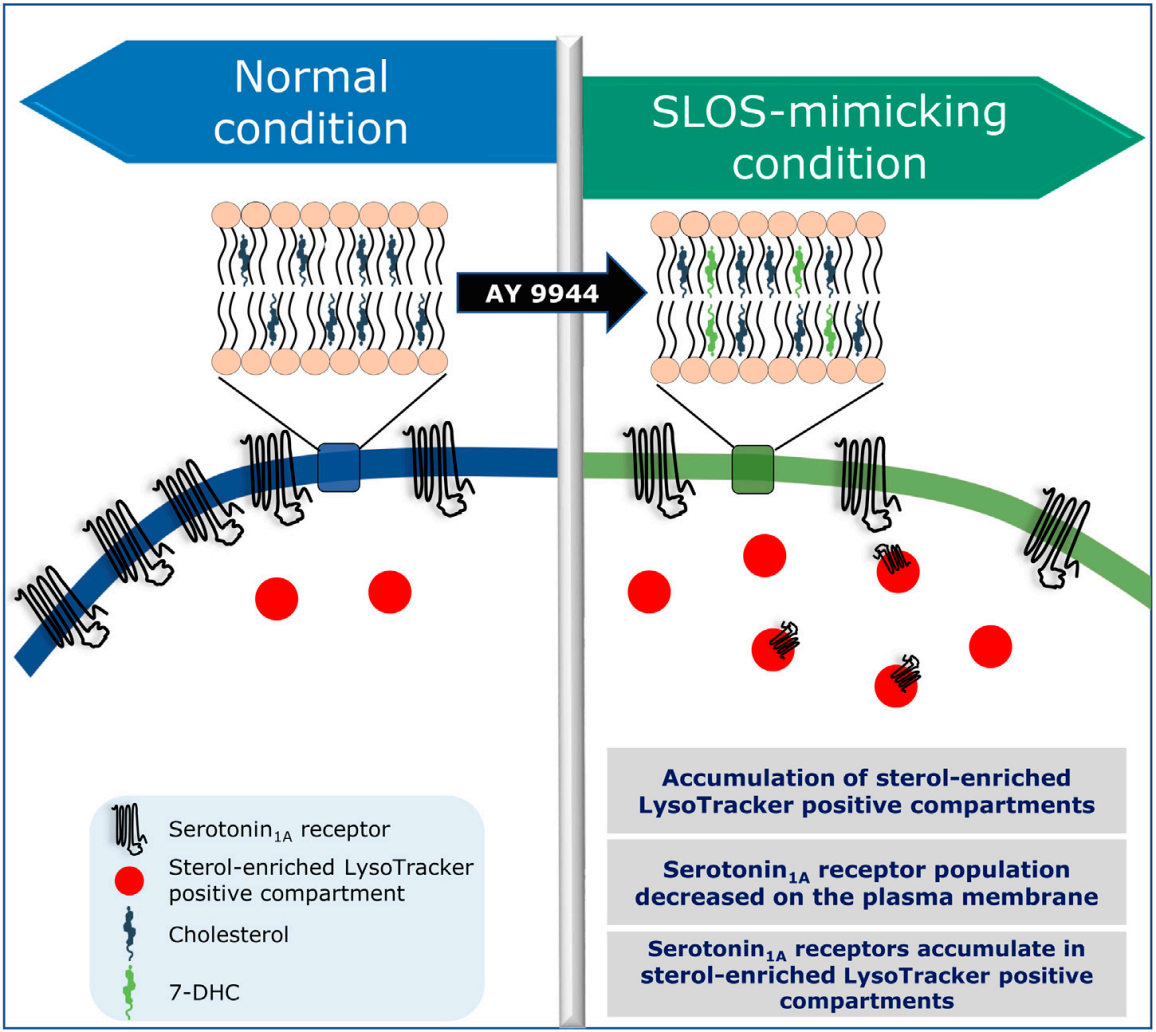
Is SLOS a trafficking disorder? A schematic representation showing altered localization and trafficking defects of the serotonin_1A_ receptor in a cellular model of SLOS. Adapted and modified with permission from Sharma et al., 2021. See text for more details.

As mentioned above, we have reported accumulation of sterol-enriched LysoTracker positive compartments in a cellular model of SLOS (Sharma et al., 2021; see Figure 6). Similar accumulation of lysosomal inclusion bodies has previously been reported in fibroblasts from SLOS patients (Wassif et al., 2002). These observations highlight the importance of altered cholesterol trafficking in SLOS. Culturing SLOS fibroblasts *in vitro* show that the accumulation of 7-DHC in SLOS led to lysosomal storage of cholesterol, sphingomyelin, and multiple glycosphingolipids, all of which are hallmarks of lysosomal storage disorders such as Niemann-Pick disease type C1 (NPC1) (Platt et al., 2014). The observation that SLOS involves a secondary defect, also observed in lysosomal storage disorders, suggest that therapies and drugs recommended for lysosomal storage disorders could be potential therapies for SLOS. In order to treat lysosomal storage disorders, a clinically approved drug known as miglustat (FDA approved as Zavesca^®^) is used (Patterson et al., 2007; Platt and Jeyakumar, 2008). Miglustat is an orally administered imino sugar drug which inhibits glucosylceramide synthase, the enzyme that catalyzes the first step in glycosphingolipid biosynthesis. Since miglustat can cross the blood–brain barrier, it has the potential to treat abnormalities associated with central nervous system. Importantly, *in vitro* treatment of SLOS cells with miglustat normalized cholesterol trafficking, with cholesterol being delivered to the ER (Platt et al., 2014). So far as SLOS is concerned, could miglustat help in correcting trafficking defects associated with GPCRs such as the serotonin_1A_ receptor in SLOS-like condition, constitute an interesting question. However, misglustat is relatively costly and this could be a limiting factor in its usage (Gutić et al., 2022).

## Epilogue

We started this review with cholesterol biosynthesis and the Bloch hypothesis. Looking back, the discovery of SLOS (Smith et al., 1964) and discovery of cholesterol biosynthesis by Konrad Bloch (Bloch, 1965) happened in similar times, although unraveling of the etiology of SLOS at the genetic level took a long time (Irons et al., 1993; Tint et al., 1994). Coincidentally, Konrad Bloch authored an article titled “Evolutionary Perfection of a Small Molecule” in a book of essays edited by him in 1994 (Bloch, 1994) where he wrote: “*In the words of Aristotle’s Politics, “Nature is the end, and what each thing is when fully developed we call ‘Nature.’” Not only genes but also small molecules changed in the course of evolution. The example cited here is the evolving structure of the sterol molecule. Along with structural changes, the functions of sterols improved and diversified....”* (Vance and Goldfine, 2002). SLOS is a prototypical example of a disease that is a consequence of Bloch hypothesis.

Since SLOS is associated with neurological malfunction, exploring the function and trafficking of neuronal receptors and their interaction with membrane lipids assume relevance. Mistrafficking of GPCRs has been implicated in diseases such as nephrogenetic diabetes insipidus, retinitis pigmentosa and cancer (Bernier et al., 2004; Dorsam and Gutkind, 2007; Hollingsworth and Gross, 2012). Recent work from our group has shown that statin-induced chronic cholesterol depletion affects the mechanism of endocytosis and the intracellular trafficking of the serotonin_1A_ receptor (Kumar and Chattopadhyay, 2020). Understanding the role of cellular lipids determining the fate of membrane receptor localization and trafficking could help in gaining novel insights into the etiology of disorders where cellular lipid composition and content is altered.
